# Author Response: The Distribution of True Visual Field Progression Rates in Glaucoma

**DOI:** 10.1167/tvst.13.12.20

**Published:** 2024-12-13

**Authors:** Giovanni Montesano, David P. Crabb, David M. Wright, Alessandro Rabiolo, Giovanni Ometto, David F. Garway-Heath

**Affiliations:** 1 City, University of London, Optometry and Visual Sciences, London, United Kingdom; 2 NIHR Biomedical Research Centre, Moorfields Eye Hospital NHS Foundation Trust and UCL Institute of Ophthalmology, London, United Kingdom; 3 Centre for Public Health, Queen's University Belfast, Royal Victoria Hospital, Belfast, Northern Ireland; 4 Department of Health Sciences, Università del Piemonte Orientale “A. Avogadro”, Novara, Italy; 5 Eye Clinic, University Hospital Maggiore della Carità, Novara, Italy. e-mail: giovmontesano@gmail.com

We greatly thank Prof. Anderson for his interest in our work and for giving us the opportunity to address these important and valid points.^1^ As correctly noted in his letter, our calculations assumed that the “true” rate of mean deviation (MD) could only assume negative or zero values. This reflects the belief that visual field (VF) loss from glaucoma can only be slowed down but not reversed (i.e., it cannot truly improve). However, the MD is an age-corrected metric,^2^ and as such, it applies a correction based on the average VF sensitivity expected for different age groups. As noted by Prof. Anderson, individual normal age-related VF decline could be faster or slower than expected. This could result in overcorrection of aging for some individuals, generating “true” positive slopes for metrics like the MD. Our assumption of no “true” improvement should, however, hold for uncorrected metrics, such as mean sensitivity (MS) of the VF.

To test this, we have recalculated our progression model for MS, using the same methodology described in our article for MD.^3^ The results are reported below (Figure and Table). The average rate of progression was 0.023 dB/year faster for MS compared to MD. This was expected, although this value is smaller than the commonly reported average age decline (−0.06 dB/year^4^). When analyzing the individual components of the ex-Gaussian distribution, we found that age correction mostly affected the estimated mean for the Gaussian component (identified as the effect of “learning” in our article), which was 0.037 dB/year smaller than that calculated for MD but still significantly different from zero. The estimated “true” rate of progression was instead very similar, changing by approximately 3.5%. This suggests that the mean of the Gaussian component is capturing both the effect of perimetric “learning” and some overcorrection of aging from the MD. This smaller initial learning effect also affected the estimated duration of learning, which became not significantly different from zero, on average, at the sixth test (0.015 [−0.014, 0.048] dB/year), as opposed to the seventh test for MD. It should be noted that these changes have no effect on the power calculations presented in our article for a randomized clinical trial based on MD, because any age overcorrection (like learning) would apply equally to both arms of the trial.

**Figure.**
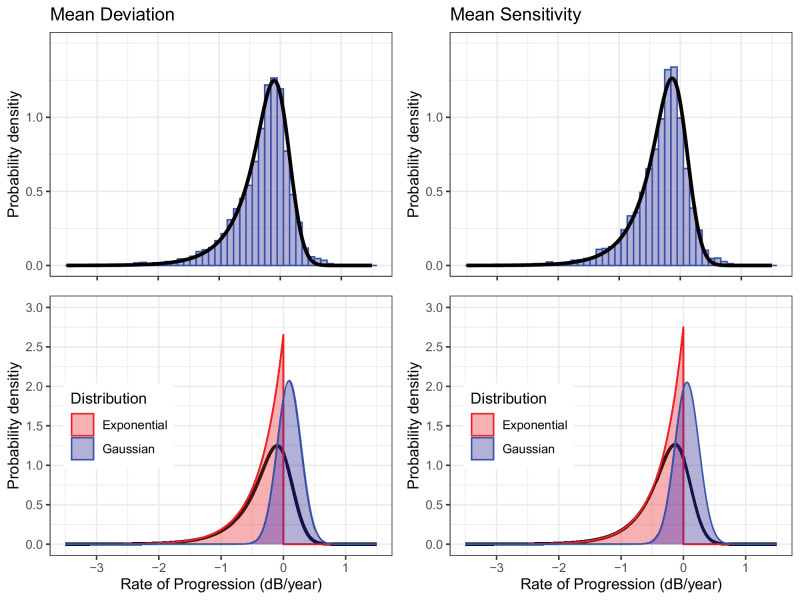
Distribution of the observed rates of progression for mean deviation (*left*) and mean sensitivity (*right*). The *black outline* represents the ex-Gaussian distribution estimated from either metric. Note that the Gaussian component is closer to 0 dB/year for the mean sensitivity.

**Table.** Parameters Estimated for the Ex-Gaussian Model for the Rate of Progression of Mean Deviation and Mean Sensitivity

	Mean Deviation	Mean Sensitivity
Exponential mean (dB/year)	−0.377 [−0.396, −0.359]	−0.364 [−0.382, −0.345]
Gaussian mean (dB/year)	0.094 [0.080, 0.109]	0.057 [0.042, 0.072]
Sample mean (dB/year)	−0.283 [−0.299, −0.268]	−0.306 [−0.321, −0.292]

The sample mean is the sum of the exponential and Gaussian mean (i.e., the average observed rate of progression).

In conclusion, we agree with Prof. Anderson that the MD, despite its widespread use, can induce distortions in the estimated rate of progression and does not strictly conform to our assumption of negative rates. However, the overcorrection of aging is, in practice, “absorbed” by the Gaussian component of our ex-Gaussian model, with minimal effect on the estimated true rate of progression.
